# A comparison of EEG encoding models using audiovisual stimuli and their unimodal counterparts

**DOI:** 10.1371/journal.pcbi.1012433

**Published:** 2024-09-09

**Authors:** Maansi Desai, Alyssa M. Field, Liberty S. Hamilton

**Affiliations:** 1 Department of Speech, Language, and Hearing Sciences, Moody College of Communication, The University of Texas at Austin, Austin, Texas, United States of America; 2 Department of Neurology, Dell Medical School, The University of Texas at Austin, Austin, Texas, United States of America; Ghent University, BELGIUM

## Abstract

Communication in the real world is inherently multimodal. When having a conversation, typically sighted and hearing people use both auditory and visual cues to understand one another. For example, objects may make sounds as they move in space, or we may use the movement of a person’s mouth to better understand what they are saying in a noisy environment. Still, many neuroscience experiments rely on unimodal stimuli to understand encoding of sensory features in the brain. The extent to which visual information may influence encoding of auditory information and vice versa in natural environments is thus unclear. Here, we addressed this question by recording scalp electroencephalography (EEG) in 11 subjects as they listened to and watched movie trailers in audiovisual (AV), visual (V) only, and audio (A) only conditions. We then fit linear encoding models that described the relationship between the brain responses and the acoustic, phonetic, and visual information in the stimuli. We also compared whether auditory and visual feature tuning was the same when stimuli were presented in the original AV format versus when visual or auditory information was removed. In these stimuli, visual and auditory information was relatively uncorrelated, and included spoken narration over a scene as well as animated or live-action characters talking with and without their face visible. For this stimulus, we found that auditory feature tuning was similar in the AV and A-only conditions, and similarly, tuning for visual information was similar when stimuli were presented with the audio present (AV) and when the audio was removed (V only). In a cross prediction analysis, we investigated whether models trained on AV data predicted responses to A or V only test data similarly to models trained on unimodal data. Overall, prediction performance using AV training and V test sets was similar to using V training and V test sets, suggesting that the auditory information has a relatively smaller effect on EEG. In contrast, prediction performance using AV training and A only test set was slightly worse than using matching A only training and A only test sets. This suggests the visual information has a stronger influence on EEG, though this makes no qualitative difference in the derived feature tuning. In effect, our results show that researchers may benefit from the richness of multimodal datasets, which can then be used to answer more than one research question.

## Author summary

An increasing number of studies employ naturalistic audiovisual stimuli to uncover the neural tracking of sensory information, but how these may differ from more traditional unimodal paradigms is not well understood. We fit encoding models to predict EEG responses to auditory features (phonological features, the acoustic envelope, pitch) and visual features (Gabor wavelet and scene cuts) using movie trailers presented in audiovisual, audio only, and visual only conditions. We found that presenting an audiovisual stimulus resulted in similar encoding model weights to those derived from auditory or visual only stimuli. However, researchers should be aware of strong influences of visual evoked potentials on the ongoing EEG if studying speech perception in the brain using audiovisual stimuli like those described here. Overall, live action and animated movie trailers that do not solely rely on face information can be used as naturalistic audiovisual stimuli to better understand neural encoding of speech or visual information.

## Introduction

Traditional approaches to understanding brain processing of speech have focused on isolated phonemes, words, or various forms of discrete or isolated sound stimuli (e.g. clicks, chirps, pure tones) presented in controlled laboratory settings. With single word stimuli, researchers have shown that visual information can supplement auditory information in noisy settings, but these stimuli were tightly controlled to investigate the potential influence of lipreading [1]. In recent years, there has been growing interest in the use of naturalistic stimuli in speech research [2–5]. Naturalistic stimuli refer to speech samples that are closer to real-world language use, such as continuous speech or conversational exchanges, rather than artificial, isolated words or sounds. This approach aims to capture the richness and complexity of human language and to better understand how speech is processed in our daily environment. Recently, studies have used continuous speech sentences [6–10] and audiobooks [11–15] to investigate speech encoding in the brain. These studies have found that neural activity measured through EEG is correlated with acoustic and linguistic stimuli, a phenomenon referred to as neural speech tracking. This approach can also be used to describe auditory feature selectivity, such as responses to specific phonological features like fricatives (e.g. /f/, /sh/, /s/, /v/, /z/, /zh/) or low back vowels (e.g /aa/), or specific responses to high frequency or low frequency spectral content in sounds [8–10, 13, 16]. However, many of these studies still use stimuli presented only through the auditory modality.

Earlier studies incorporating audiovisual stimuli have shown that visual information in the form of lip-reading modulates auditory perception, especially in challenging listening situations [1, 17, 18] and for listeners with hearing loss [19–21]. Using magnetoencephalography (MEG), Haider and colleagues demonstrated that obscuring the mouth impaired the brain’s ability to track [20] and reconstruct [21] speech. In the 2024 study, Haider et al. were curious to study how the use of face masks affected the way the brain processes audiovisual versus only auditory information. They presented stories in an audiovisual format where the speaker was wearing a face mask in one condition and was not in another. In both conditions of the experimental design, the masked and unmasked audiovisual information was contrasted with just an audio-only version of the story stimuli. Linear encoding models were fit to predict neural responses of the spectrogram and lip-reading information. Results from this study found that adding lip reading information to the audiovisual condition improves the overall model prediction accuracy, particularly in the occipital scalp sensors. The presence of visual information to track speech content, particularly in situations when a speaker is wearing a mask, improves the neural tracking of speech, and the brain seems to depend on the speaker’s lips to better comprehend acoustic information.

Others have investigated modulation of visual motion perception by congruent audiovisual stimulation during adaptation of the concept of visual motion aftereffect (or the experience of a visual illusion after seeing moving object or scene for a period of time), demonstrating a bidirectional influence between visual and auditory motion [22]. This group found that when auditory and visual information was presented congruently, a motion aftereffect was stronger and lasted longer when there was a visual and auditory stimulus moving in the same direction compared to just being presented with the unimodal visual motion stimulus. Studies by Crosse et al. 2015 and Crosse et al. 2016 provided insight into how congruency between audio and visual speech enhances cortical entrainment and improves speech understanding in noisy environments [17, 23]. In both papers, the stimulus was a video of a male talker speaking conversationally, presented with the head, shoulders, and chest in frame. In one of these studies [23], they varied the congruence of the video and audio information, and in the other [17], they added background noise. This group found that the visual input heavily contributed to the brain’s ability to track speech in noisy listening conditions compared to speech presented in isolation, and that congruent visual information enhances speech tracking in non-noisy environments. Finally, our understanding of audiovisual processing has been expanded by work that examined delta-theta phase modulation in MEG participants who viewed naturalistic audiovisual movie clips [24]. Here, the authors found that the auditory and visual cortices can continuously track and discriminate the unimodal information presented in naturalistic audiovisual stimuli. Taken together, these studies have provided foundational evidence in the intersection of the neural tracking of audiovisual stimuli, the importance of both sensory modalities playing a role in perception, as well as the use of naturalistic stimuli to better understand audiovisual integration.

In contrast to prior work using naturalistic audio and visual recordings [17, 20, 23, 25, 26], we used children’s movie trailers. These movie trailers included live-action as well as animated or cartoon figures from well-known movies including Inside Out, Paddington 2, Ferdinand, Deep, Pele, The Little Prince, and Big Hero 6. A motivation of using these movie trailers involves the desire to collect more engaging, ecologically valid, and acoustically rich stimuli when working with pediatric or clinical populations. The movie trailers contained speech, music, and various background sounds. In a prior EEG study, we demonstrated that it was possible to derive receptive fields from these audiovisual stimuli that are similar to more traditionally controlled listening paradigms [9]. While our previous work examined the role of visual information on auditory encoding, the movie trailers were presented only in their original audiovisual format. In the current study, we separated the stimuli into auditory and visual counterparts and presented EEG participants the original movie trailers in their audiovisual form, the movie trailers with only the visual information, and the movie trailers with only the auditory information. In this way, we could investigate how visual information influences auditory feature selectivity and vice versa and how this differs to auditory or visual information presented in isolation. Importantly, these stimuli were not designed to have a specific relationship between the auditory and visual features, but instead maintained their natural relationship.

We used EEG to record neural responses to speech and visual information in children’s movie trailers under three different conditions: audiovisual (AV), visual only (V), and audio only (A). Our first goal was to determine if auditory information was similarly represented when participants watched AV movies versus listening to the movie audio only (A). Then, we wanted to determine if neural responses to visual features were similarly represented in AV versus V conditions. In our previous work [9], we found that the visual information explained a large proportion of the overall explained variance, however, this was largely independent of auditory information. Given our prior work showing that encoding models fit on audiovisual movie trailers generalize to audio-only sentence stimuli [9], we hypothesized that the brain’s response to acoustic and phonetic features in AV would be similar to brain responses in A in the movie trailer stimulus set. This would mean that it would still be possible to use an audiovisual stimulus to investigate encoding of auditory features in the brain.

Using more engaging audiovisual stimuli may be fruitful for studying individuals who cannot tolerate lengthy and tedious tasks [9, 27]. Because of the low signal-to-noise ratio of EEG, long experimental sessions are often required when investigating brain processing of speech. For children and clinical populations, long experimental sessions may not be tolerable at all if the tasks are also considered to be boring or unpleasant. Thus, our research is partially motivated by attempts to create tasks that will maximize data quality and amount of data that can be collected for an experimental paradigm [27].

The purpose of the current study is to understand how information in one sensory modality (auditory or visual) is encoded by the brain during audiovisual movie watching versus unimodal presentation of these stimuli during EEG recordings. The results from this study will provide practical information for interpreting the results of experiments using one type of naturalistic multimodal stimulus (animated movie trailers), while allowing experimenters to interpret multimodal or unimodal information depending on the research question at hand.

## Materials and methods

### Ethics statement

All experimental procedures were approved by The University of Texas at Austin Institutional Review Board. All participants gave written informed consent to participate.

### Data

#### Participants

11 native-English participants with normal hearing (5M, 5F, 1 N/B, age 18-31, mean age: 25.6 ± 3.95 years) were recruited to participate. Pure tone thresholds (250-8000 Hz) and speech-in-noise hearing tests (QuickSIN, Interacoustics) were administered to ensure that the participants had normal hearing (< 25 dB HL thresholds for pure tone and < 3 dB SNR loss for QuickSIN). All participants had normal or corrected-to-normal vision.

#### Experimental design

EEG participants listened to and watched children’s movie trailers for approximately 1 hour, taken from a previously published dataset (https://osf.io/p7qy8/) [9]. These stimuli contained overlapping speech, music, background sounds, and visual information. The movie trailer stimuli were hand-transcribed to reflect the onset and offset of phoneme and word-level boundaries using ELAN (https://archive.mpi.nl/tla/elan) and a modified version of the Penn Phonetics forced aligner, FAVE align (https://zenodo.org/record/9846). Timings were manually corrected using Praat by four raters to ensure reliability. In addition to the linguistic information, the onset and offset of scene changes was marked. Finally, the raters transcribed speech-related mouth movement information, including when the face of a speaker could be seen and was congruent with the speech audio (23.8% of time samples), when the face was not visible despite ongoing speech information (66.4% of time samples), and when there was no speech (9.8% of time samples).

During the task, all EEG participants listened to and watched eight movie trailers in an audiovisual condition (AV: listening and watching), a visual only condition (V: only watching with no audio), and a listening only condition (A: only listening to the audio and staring at a fixation cross). AV, V, and A trials were presented in pseudo-random order. The task was presented through an iPad running custom software written in Swift (version 5.3.2; https://developer.apple.com/swift/). The iPad was placed on a table at precisely 23 inches from the participant, measured from the nasion to the top of the iPad. Videos were rendered at 1280 × 720-pixel movies at 24 frames/s. The audio was sampled at 44.1 kHz. Each movie trailer for AV, V, and A conditions was presented once.

#### Data acquisition

High-density EEG data were collected using a standard 64-channel montage (EasyCap) at a sampling rate of 25 kHz using the BrainVision actiCHamp system (Brain Products). Impedance for all channels was kept below 15 *k*Ω. An additional two channels were used to measure vertical and horizontal electrooculography (EOG) to remove ocular artifacts during preprocessing. The audio was recorded as a separate channel in addition to the EEG data from the task and was synchronized with the neural data using a StimTrak stimulus processor (Brain Products). The visual only condition contained a short notification sound which was used to synchronize the onset of the stimulus with the EEG data.

#### EEG preprocessing

EEG and EOG electrode time courses were downsampled to 128 Hz using the BrainVision Analyzer 2.1 software. Subsequent preprocessing steps were conducted offline using MNE-Python [28]. EEG channels were re-referenced to the average of the mastoid channels (TP9 and TP10) and then notch filtered at 60 Hz to remove line noise. The data were bandpass filtered between 1-15 Hz using a zero-phase, noncausal bandpass FIR (finite impulse response) filter (Hamming window, 0.0194; passband ripple with 53 dB stopband attenuation, −6 dB falloff). Manual artifact rejection was conducted to remove large movement artifacts, and no more than 10% of the data were removed. Independent component analysis (ICA) with 64 components was used to remove blinks and saccades. We implemented a match filter procedure [29] to convolve the stimulus waveform with the recorded audio from the EEG experiment using custom python scripts. Here, the code identified the waveform of the stimulus presented in the neural recording to match the stimulus from the original sound wavefile for the AV and A-only conditions. In the case of the V-only condition, a unique notification sound such as a beep or chime was presented at the onset of the trailer in order for us to implement the match filter procedure and precisely align the onset of the movie trailers with the neural recording. Finally, EEG data were epoched to the onset and offset of the acoustic stimuli (for AV and A conditions) and the short notification sounds (V only). Once epoched, the timings of the acoustic and phonetic information from the Praat textgrids were used for further analysis.

#### Auditory and visual feature extraction

**Fig 1:**
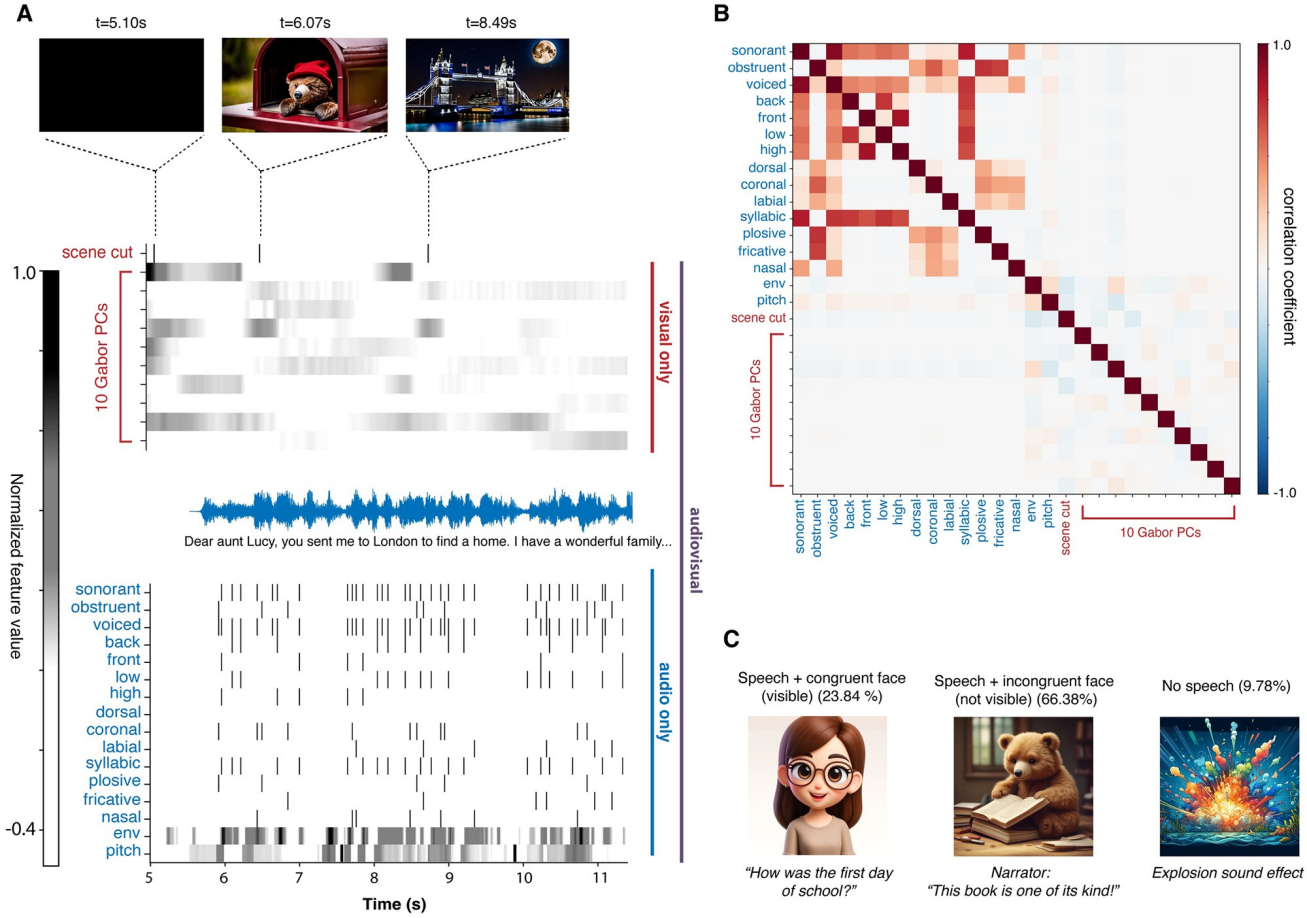
Feature schematic showing stimulus input for encoding model. A) We fit models predicting neural activity from auditory (phonological features, the acoustic envelope, and pitch) and visual (scene cuts and Gabor wavelet filters) features from movie trailer stimuli presented in an audiovisual format, audio only format, or visual only format. B) Correlation between features across all movie trailers. Higher correlations were observed between phonological features (for example, sonorant+voiced), but overall other auditory and visual features were uncorrelated. C) Example of speaker congruent and incongruent visual scenes in the movie trailer stimuli. The percentage of time for each of these event types across all movie trailers are shown in the title above. Example images similar to those shown in the experiment were generated using ChatGPT DALL-E (https://www.dall-efree.com/). Original images presented to participants are not shown here due to copyright restrictions.

We extracted both auditory and visual features from the movie trailers, examples of which are shown in Fig 1. For auditory features, we extracted the acoustic envelope, pitch, and phonological features. For phonological features, we created a binary matrix to indicate the timing of all phonemes based on place and manner of articulation for each movie trailer (e.g. sonorant, obstruent, fricative, etc.) [9, 10]. The matrix contained a 1 at the onset of the phonological feature and 0 for all other times. The acoustic envelope of the stimuli was extracted using the Hilbert-transform of the waveform followed by a low-pass filter (third-order Butterworth filter; cutoff frequency, 25 Hz) and downsampling to 128 Hz. Lastly, the absolute pitch was calculated using an autocorrelation method implemented in Parselmouth [30], a python package that interfaces with Praat [31], with a 7.8 ms window (1/128 Hz) and 50-300 Hz F0 range.

While the movie trailers are acoustically rich, these stimuli also contain visual information. To investigate the influence of these visual features on audiovisual speech encoding, we fit encoding models using scene cuts and Gabor wavelet filters in addition to the acoustic features above. Scene cut information was taken from Praat textgrids, which contained hand-annotated onsets of scene changes. Gabor wavelet filters were calculated using a nonlinear Gabor motion energy filter bank [32] to capture varying visual motion for every frame of each movie trailer. These procedures were identical to those used in [9] and were adapted from [32], but are also described below. We first converted each frame of the movie from 720 x 1280 pixels to 1280 x 1280 pixels by zero padding with black pixels at the top and bottom, then downsampled to 96 x 96 pixels. Next, the frames were converted to grayscale by converting RGB to L*A*B color space and retaining the luminance channel. These grayscale movies were then decomposed into 2,139 Gabor wavelet filters, which are 3D spatiotemporal sinusoids multiplied by a 3D spatiotemporal Gaussian envelope. We used five spatial frequencies, log spaced from 1.5 to 24 cycles/image, three temporal frequencies (0, 1.33, and 2.667 Hz), and eight directions (0–315° in 45° steps). We calculated velocities over 10 frames of the movie at a time. We included zero temporal frequency filters at 0°, 45°, 90°, and 135°, and one zero spatial frequency filter. The filters are positioned on each frame of the movie. Adjacent Gabor wavelets were separated by four standard deviations of the spatial Gaussian envelope. Each filter was also computed at two quadratic phases (0° and 90°), as in the study by Nishimoto et al. (2011). The Gabor features were then log transformed to scale down very large values. Finally, to reduce the complexity of these features and make the feature space more comparable to the acoustic features, we decomposed these 2,139 filters into 10 principal components to reduce the dimensionality of the feature matrix. In our prior work, we showed that these 10 principal components were sufficient to capture variance in the EEG data [9].

#### Linear encoding models

Encoding models (known as multivariate temporal response functions or temporal receptive fields (mTRFs), or forward models) are a method for describing the statistical relationship between a stimulus (e.g. visual or auditory feature representations) and neural response [8–10, 13, 33–35]. We fit mTRF models to all 64 channels from all EEG participants, using separate auditory or visual feature models (Eq 1).

$$\hat{y}\left(t,n\right)=\sum_{f}\sum_{\tau }w\left(f,\tau ,n\right)s\left(f,t-\tau \right)+\varepsilon \left(t,n\right)$$ (1)

$\hat{y}\left(t,n\right)$ represents the neural response for each EEG channel, *n*, at time, *t*. *w*(*f*, *τ*, *n*) is a matrix of weights fit from the model for a given feature representation (*f*), at each channel (*n*), and time delay (*τ*). Weights were fit using ridge regression on a subset of training data (Eq 2).

$${\hat{w}}_{ridge}={\left(S^{⊤}S+\lambda I\right)}^{-1}S^{⊤}Y$$ (2)

The regularization parameter (λ) was estimated using a separate cross-validation procedure, where we tested 15 values of λ between 10^2^ and 10^8^. This was performed for 20 iterations of portions of the training set. The time delay (*τ*) for each model fit was between 0 and 600 ms. *s*(*f*, *t* − *τ*) is the stimulus input used to fit the encoding model for a given feature representation for the specified time delay. *ε*(*t*, *n*) are the residual errors for the linear regression. The test set data consisted of the neural response and stimulus information from the same two movie trailers (Inside Out and Paddington) for each respective condition (AV, V, A). The remaining 6 trailers were used as training data. For each encoding model using separate auditory or visual features, the performance was evaluated by calculating the correlation (*r*) between the predicted EEG and the actual held out EEG. The significance of each model was assessed using a subsampling shuffle procedure. Briefly, the relationship between the stimuli and neural responses was broken by shuffling the stimulus in chunks of 2 seconds and calculating the model based on the randomized data. The correlation between the predicted and actual EEG for this shuffled model was then compared to the original model 100 times to compute a p-value. Models were considered significant for a p-value of *p* < 0.05.

#### Multimodal and unimodal model comparisons

**Fig 2:**
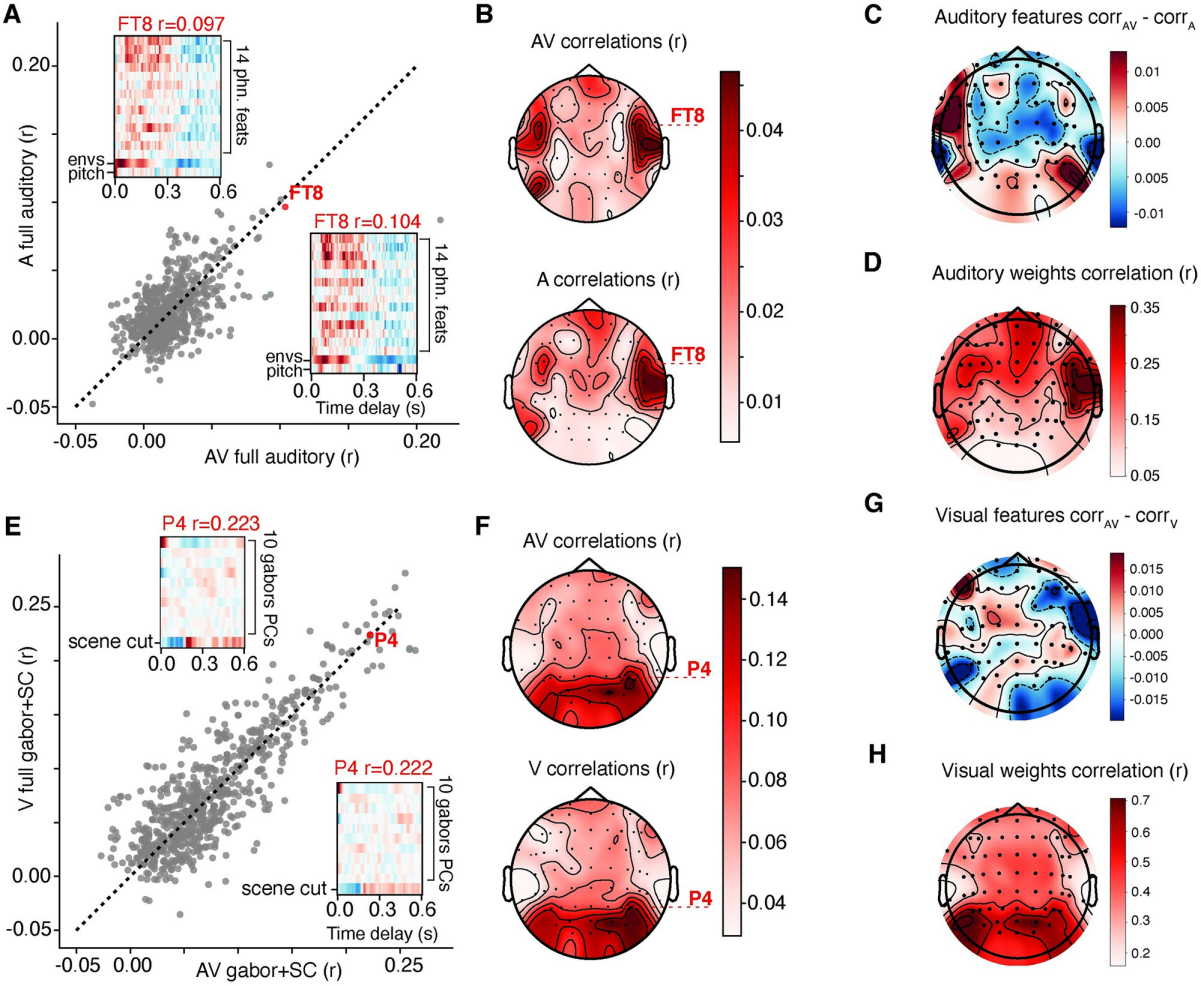
Comparison between auditory and visual feature models to predict EEG. A) Scatter plot showing the model comparison (*r*-value) between AV and A conditions, where the original (AV) movie trailer was present, or an audio only version (A). In both cases, the same set of auditory features were used to predict the data. Each gray dot represents the encoding model performance in a single channel in a single participant. Channel FT8 is shown for subject MT0033 with the corresponding weight matrices for each condition (A condition: top left, AV condition: bottom right) and associated correlation value for the channel. B) Grand average correlation values for AV and A condition plotted on topographical map and averaged across all participants (*n* = 11). Topography of selective channels was similar for AV and A. C) Average difference in prediction performance for AV and A for all participants. D) Average correlation between weights between acoustic and linguistic features from A and AV across all participants. The receptive field structure was similar over temporal, frontal, and central sensors. E) Scatter plot showing the model comparison (*r*-value) between AV and V conditions, where the original (AV) movie trailer was present, or a visual only version (V). Visual feature model used a combination of 10 Gabor wavelet filter principal components (PCs) and scene cut (SC) information. Each gray dot represents the encoding model performance in a single channel in a single participant. An example channel, P4, is shown for subject MT0029 with the corresponding weight matrices for each condition and associated model performance (correlation value) for the channel. F) Grand average correlation values for AV and V condition plotted on topographical map and averaged across all participants (*n* = 11). Similar spatial distribution of good model performance was observed regardless of condition. G) Same as C for visual feature models fitted with AV and V data. H) Average correlation between weights between visual features for V and AV across all participants. Receptive field structure was most similar over occipital sensors.

We compared the performance of models trained on multimodal audiovisual data to the performance of models trained on unimodal (auditory only or visual only) stimulus presentations. In addition, we compared the structure of the weights and derived feature tuning across EEG channels when using these different stimuli. Topographical maps were plotted to show the spatial distribution of encoding model performance for each condition type, as well as the correlation between the weight matrices using different training sets (e.g. Fig 2D and 2H).

To test for significant differences between the unimodal and multimodal models, we fit linear mixed effects models with the model type (A versus AV, V versus AV, predicting V from V versus V from AV, and predicting A from A versus A from AV), region of interest (temporal, frontal, occipital, central, or parietal, based on scalp EEG channel), and their interaction as fixed effects, and the subject ID as a random effect. These models were implemented in R using the library lmerTest [36] and the equation: lmer(“correlation ∼ condition * roi + (1|subject)”) where *correlation* was the correlation between actual and predicted EEG, modeled by the fixed effects of *condition* as either AV, V, or A and *roi* as the region of interest of sensors across the scalp as central, parietal, frontal, occipital, and temporal. We also considered the interaction of condition and ROI, since different stimulus conditions may influence the prediction performance differently depending on ROI (for example—visual information might have a stronger effect on occipital electrodes). Inter-subject variability was controlled using subject as a random effect.

#### Cross prediction analysis

Part of our goal in this study was to determine whether stimulus selectivity was similar in unimodal (auditory or visual only) conditions compared to the audiovisual condition. To determine whether receptive field models generalized across condition type, we compared performance of models based on the audiovisual stimulus-derived receptive fields or the auditory only stimulus-derived receptive fields. We also performed the same analysis to compare predictions of neural responses to visual information when using the audiovisual data versus the visual only data. If the audiovisual stimulus does not significantly affect the structure of receptive fields, we should see prediction performance that is similar for unimodal versus multimodal conditions.

When predicting auditory responses from audiovisual weights, we used all speech features (phonological features, the acoustic envelope, and pitch). When predicting visual responses from audiovisual weights, we used all visual features (Gabor wavelet filters and scene cuts). We then used the weights calculated from the audiovisual condition as the training set to predict the neural responses of the respective speech (auditory condition) or visual (visual condition) features in the movie trailer EEG data. That is, using pre-trained models from the audiovisual condition, we predicted responses to the auditory or visual only condition as the test set. We then compared the model performance for the cross-condition analysis to the model performance for the original within-condition analysis, where training and test data came from the same condition type. The cross prediction equation for predicting A only data from mTRFs calculated from the AV training data is shown in Eq 3:

$$sEEG_{A}\left(t,n\right)=\sum_{f}\sum_{\tau }w_{AV}\left(f,\tau ,n\right)s_{A}\left(f,t-\tau \right)+\varepsilon \left(t,n\right)$$ (3)

And to calculate EEG in response to V only stimuli from mTRFs calculated from the AV condition in Eq 4:

$$sEEG_{V}\left(t,n\right)=\sum_{f}\sum_{\tau }w_{AV}\left(f,\tau ,n\right)s_{V}\left(f,t-\tau \right)+\varepsilon \left(t,n\right)$$ (4)

We used a linear mixed effects model [36] in R to test the difference between the model performance in A versus V versus AV. We tested for significant interaction between the EEG scalp channels (frontal, central, occipital, temporal, parietal) with the model type (full visual model consisting of scene cuts + Gabor wavelet filters or the full auditory model consisting of phonological features + acoustic envelope + pitch) for each condition (A, V, AV).

## Results

### Auditory and audiovisual feature representations in the brain

Encoding models provide a statistical metric to map the relationship between the brain’s response and continuous stimuli. We trained on 64-channel EEG data responses using a combination of 14 phonological features, the acoustic envelope, and pitch. These features have been used in previous work to investigate acoustic and linguistic selectivity to continuous speech stimuli [8–10, 13]. Model performance was assessed by comparing the linear correlation value (*r*) between the actual and predicted EEG for each condition (A versus AV) on held out data. We hypothesized that encoding of auditory features between A and AV would generate comparable model performance because our previous results demonstrated that the visual information from the movie trailers contributed a large amount of variance to the EEG response [9]. Model performance was similar across all subjects for both A (*r*_*max*_ = 0.13, *r*_*avg*_ = 0.02) and AV (*r*_*max*_ = 0.22, *r*_*avg*_ = 0.02) conditions (Fig 2A). Individual model performance for the full auditory model in each EEG channel in all subjects was compared for A vs. AV data, with most points falling along the unity line. The structure of the auditory feature weights for AV versus A looked similar, with broad selectivity and enhancement of voltage changes to linguistic and acoustic features for both conditions between 0.0 and 0.25 seconds post stimulus onset. Topographical maps show that the highest auditory model performance was primarily concentrated in fronto-temporal regions of the scalp for both AV and A models (Fig 2B). Using linear mixed effects models to test the difference in model performance for AV versus A, we found there was no main effect of the condition (*estimate* = −3.027 × 10^−4^, *df* = 1388, *p* = 0.78). However, we observed significant differences in model performance by region of interest (ROI) (frontal: *estimate* = 2.86 × 10^−3^, *df* = 1388, *p* = 0.0189; central: *estimate* = 2.008 × 10^−3^, *df* = 1388, *p* = 0.16; parietal: *estimate* = −6.595 × 10^−3^, *df* = 1388, *p* = 9.68 × 10^−5^, occipital: *estimate* = −8.1055 × 10^−3^, *df* = 1388, *p* = 4.94 × 10^−6^; temporal: *estimate* = 9.831 × 10^−3^, *df* = 1388, *p* = 1.62 × 10^−9^). This indicates, as expected, that frontal and temporal channels showed the highest degree of selectivity for auditory information, regardless of how the stimulus was presented. Finally, there was only a significant interaction between AV and frontal ROI (*estimate* = −5.118 × 10^−3^, *df* = 1388, *p* = 0.01). The average difference in model performance across all participants for A versus AV showed a small difference that was distributed across the scalp (Fig 2C). On the other hand, the weight matrices for both models were highly correlated in the temporal electrodes, indicating similar responses to auditory features regardless of A vs. AV condition (Fig 2D).

### Visual feature representations in the brain

Our previous analysis showed that auditory feature encoding was comparable between AV and A only conditions. However, the visual features themselves are a prominent perceptual experience when engaging with movie trailers. So how much does auditory information impact visual encoding in these stimuli, if at all? Previous work using the same stimuli demonstrated that the addition of visual information contributed to a significant portion of variance explained, but that this variance was mostly independent of that explained by the auditory and phonological features [9]. To better understand how auditory signals impact visual feature encoding models, we next compared model performance when movie trailers were presented without any audio and compared this to the audiovisual model. We trained models for all 64 EEG channels across all participants using a combination of Gabor wavelet features and a binary feature vector of scene cut information [9, 32] as our visual stimulus.

Due to the relative independence of auditory and visual features (Fig 1), we hypothesized that visual information should be similarly represented in the AV and V only condition. We compared correlation (*r*) values between actual and predicted EEG data from V and AV across all participants using 11 visual features (Gabor wavelets and scene cuts). Encoding model performance was similar for the V (*r*_*max*_ = 0.28, *r*_*avg*_ = 0.08) and AV (*r*_*max*_ = 0.26, *r*_*avg*_ = 0.08) conditions (Fig 2E). Individual channel encoding models were distributed across the unity line. The structure of the weights was visually similar especially in visually selective channels, as shown for an example single channel (P4) in one participant (MT0029) and more broadly in occipital channels (Fig 2H). In the example inset in Fig 2E, the scene cuts induce similar voltage changes across time delays in both V and AV conditions. Model performance for AV (*r* = 0.222) and V (*r* = 0.223) conditions were also highly similar for this channel. Topographical maps of the average correlation values from all subjects across all 64 EEG channels show that the highest *r*-values for visual encoding models were concentrated in occipital regions of the scalp (Fig 2F), regardless of AV or V presentation. Using linear mixed effects models to test the difference in model performance for AV versus V, we found there was no significant main effect of the condition type (*estimate* = 2.755 × 10^−3^, *df* = 1388, *p* = 0.22). On the other hand, we observed significant differences in visual model performance across ROIs (frontal: *estimate* = −2.253 × 10^−2^, *df* = 1388, *p* < 2 × 10^−16^; central: *estimate* = −1.391 × 10^−2^, *df* = 1388, *p* = 2.11 × 10^−6^; parietal: *estimate* = 2.679 × 10^−2^, *df* = 1388, *p* = 1.42 × 10^−14^; occipital: *estimate* = 4.728 × 10^−2^, *df* = 1388, *p* < 2 × 10^−16^; temporal: *estimate* = −3.763 × 10^−2^, *df* = 1388, *p* < 2 × 10^−16^). This indicates that the visual model performance is highest in occipital channels, regardless of AV vs V only presentation condition. Unlike the comparison between AV and A, there was no significant interaction between condition type and ROI.

Additionally, there was no apparent structure to the distribution of electrodes that were more responsive in either V or AV (Fig 2G). Weights from the V and AV model were most correlated in occipital channels across all participants (Fig 2H).

Given prior work showing potential auditory responses during lipreading, we wished to know whether auditory or phonological information could be inferred from the visual information, even though no sounds were presented in the V condition. Thus, we fit an encoding model using the 14 phonological features and compared model performance correlation values (*r*) between the AV and V conditions. Although sound was technically absent in the V condition, we used the timings of the phonological features from the AV condition, which would be the same. We found that models predicting responses to the inferred phonological features in the V condition performed below chance (*r*_*max*_ = 0.088, *r*_*avg*_ = −0.0015), with no significant difference between this model and the null shuffle model (*p* > 0.05). On the other hand, prediction performance was much higher in the actual AV condition (*r*_*max*_ = 0.11, *r*_*avg*_ = 0.017) and A condition (*r*_*max*_ = 0.13, *r*_*avg*_ = 0.019). These results demonstrate that phonological features are not obviously represented in the V condition compared to AV or A. This difference may be due to the relative independence of the auditory and visual information in this stimulus set (Fig 1B), unlike studies assessing lip-reading where most audiovisual information is congruent [19, 23, 37, 38]. While previous results compared auditory and visual feature encoding in AV, V, A conditions, the impact of combining specific visual and auditory information, such as congruent and incongruent mouth movement with phonological feature encoding in AV and V conditions is still unclear. Previous work has demonstrated that congruent face and lip movement enhances audio encoding in the brain [19], however, our study showed that such effects were marginal for our stimulus set. This was likely due to the relative independence of the auditory and visual information (we saw only 23.8% of time points that included congruent face and speech information, whereas 66.4% included incongruent face and speech, and 9.8% contained no speech and only music or background sound effects). Many of these movie trailers were also animated, so it is possible that these effects are weaker for animated versus real human faces speaking.

### Cross prediction analysis

In the previous analysis, we showed that prediction performance was comparable for unimodal and multimodal encoding models (Fig 2). In addition, the receptive field models derived from data in both unimodal and multimodal conditions exhibited similar selectivity. Still, this comparison does not address whether models fit on one data type (uni- or multimodal) would predict responses to the other type of dataset.

To address this, we conducted further analyses to determine whether encoding models trained using audiovisual could generalize and accurately predict neural responses in the unimodal conditions (A only or V only). The motivation behind this analysis was to test whether the presence of visual information significantly influenced predictions for the auditory modality compared to when information is presented in isolation. This would allow us to determine if auditory receptive fields or visual receptive fields derived from audiovisual information are a reliable estimate of how the brain responds in the unimodal context. This would allow researchers to consider using more naturalistic multimodal stimuli in their experiments, even if their research question is related to unimodal sensory encoding.

**Fig 3:**
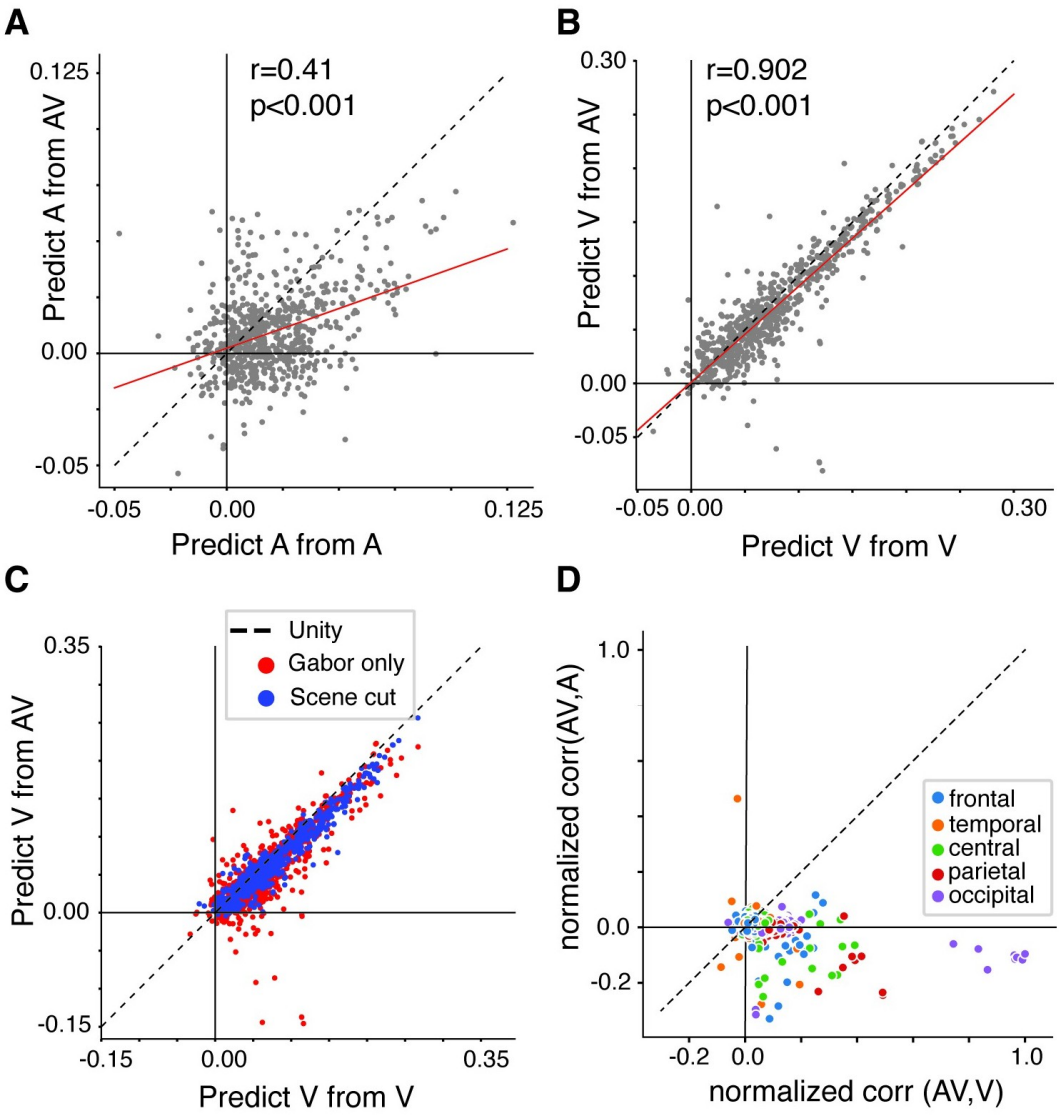
Cross prediction analysis shows that responses are generalizable between unimodal and multimodal stimulus information, with stronger generalizability for visual information compared to auditory. A) Model performance for audio-only (A) test sets with A-only training data (x-axis) or audiovisual (AV) training data (y-axis), calculated as the linear correlation between predicted and actual held out EEG test data. Features for this model included phonological features, the acoustic envelope, and pitch. Each dot represents an individual electrode for an individual EEG subject (64 channels x 11 participants). Dashed black line = unity line; red line = regression line. B) Model performance for visual-only (V) test set with V-only training data or AV training data. Features for this model included the 10 Gabor PCs and scene cuts. Similar model performance was observed for both within- and cross-condition predictions, though this relationship was stronger between V and AV. C) Model performance for comparing visual only responses using either scene cuts or only Gabor feature representations with the individual feature in the audiovisual condition. D) Normalized correlation coefficient between each EEG channel for audiovisual and visual only conditions and audiovisual and audio only conditions. Overall, single trial bandpass filtered EEG (input to the model) was more correlated between the AV and V only conditions as compared to the AV and A only conditions, suggesting a strong influence of visual information on the EEG signals.

For the cross-prediction analysis, we manipulated the training set type (uni vs. multimodal) and measured prediction performance for data from each unimodal test set. For example, we used the acoustic model weights (phonological features, pitch, and acoustic envelope) from the A only condition to predict responses to test set data from A-only and test set data from AV neural data. Similarly, we used the combination of Gabor wavelet filters and scene cuts from the V-only condition to predict responses to test set V only and AV EEG data. For both of these cross-predictions, we compared the performance of the model trained on the unimodal condition and tested on the same unimodal condition (e.g. predicting unimodal A or V from unimodal A or V training set data) or trained on the multimodal condition (e.g. predicting A or V from AV training set data). Results of this analysis are shown in Fig 3. Correlation values below the unity line indicate electrodes for which the within-condition model performance was better, and those above the unity line indicate better cross-condition performance. A regression line was plotted for both stimulus comparisons to determine how correlated the within-condition encoding model was with the cross-condition model performance. Overall, using the same condition type for training and testing (e.g., predict A test data from A training data or predict V test data from V training data) resulted in better model performance than cross-predictions (e.g. predict A test data from AV training data, or predict V test data from AV training data). However, the response to one condition (A or V) could be modeled from the AV condition and performance was significantly correlated (*r* = 0.41, *p* < 0.001 for the auditory comparisons in Fig 3A, and *r* = 0.902, *p* < 0.001 for the visual comparisons in Fig 3B). Similar to the previous results, predicting auditory responses from A-only and AV data resulted in lower correlation values (Fig 3A) compared to the predicting visual responses from V-only and AV data (Fig 3B).

In a direct comparison of within-condition and cross-condition models, linear mixed effects models demonstrated a significant difference between predicting A from A and A from AV using the full auditory model, which consisted of the combination of phonological features, acoustic envelope, and pitch (*estimate* = −4.007 × 10^−3^, *df* = 1388, *p* = 1.67 × 10^−7^). There was a significant effect of ROI on model performance (frontal: *estimate* = 2.340 × 10^−3^, *df* = 1388, *p* = 0.0056; central: *estimate* = 5.906 × 10^−3^, *df* = 1388, *p* = 3.07 × 10^−9^; parietal: *estimate* = −5.428 × 10^−3^, *df* = 1.388, *p* = 3.65 × 10^−6^; occipital: *estimate* = −5.838 × 10^−3^, *df* = 1388, *p* = 2.02 × 10^−6^; temporal: *estimate* = 3.019 × 10^−3^, *df* = 1388, *p* = 0.007). As before, this indicates better auditory model performance in temporal, frontal, and central channels regardless of training set type. There was no significant interaction between condition type and ROI.

To quantify the similarities in the cross prediction analysis when predicting V from V versus from V and AV using the full visual model (scene cuts plus Gabor wavelet filters), the linear mixed effects models showed no significant main effect of model type (*estimate* = 1.418 × 10^−3^, *df* = 1388, *p* = 0.448). As before, there was a significant effect of ROI (frontal: *estimate* = −2.189 × 10^−2^, *df* = 1388, *p* < 2 × 10^−16^; central: *estimate* = −1.739 × 10^−3^, *df* = 1388, *p* = 1.2 × 10^−12^; parietal: *estimate* = 3.861 × 10^−2^, *df* = 1388, *p* < 2 × 10^−16^; occipital: *estimate* = 3.21 × 10^−2^, *df* = 1388, *p* < 2 × 10^−16^; temporal: *estimate* = −3.143 × 10^−2^, *df* = 1388, *p* < 2 × 10^−16^). This shows that visual model performance was significantly higher in parietal and occipital channels.

Overall, testing and training using unimodal conditions (A from A and V from V) elicited better model performance. However, responses were still able to be derived from the multimodal (AV) condition. In previous work, we found that the model performance for a visual feature model was greater than models using acoustic information when using the movie trailer in its original multimodal form [9]. The results here (Fig 3A and 3B) corroborate such findings for unimodal stimuli. Still, we found that even when using the multimodal (AV) condition data, it was still possible to encode neural responses to A or V-only stimulus information. This suggests that audiovisual stimuli provide generalizable results to unimodal stimuli, with the added benefit of providing a rich source of information for asking multiple research questions.

While we found that responses were generalizable between unimodal and multimodal stimuli, we found that cross-prediction performance was better for visual models compared to auditory. We hypothesized that the correlation from the cross-prediction analysis for the predict V from V versus V from AV may arise from highly salient scene cut information, which could contribute to a larger dynamic visual response. To test if the model performance from the visual information differed based on feature representation, we conducted a cross prediction analysis using the Gabor only or scene cut only model from the V only condition and tested on the same visual feature in the AV condition (Fig 3C). We found that the scene cut information was concentrated along the unity line, suggesting that the scene cut information was equally modeled in the V-only and AV conditions. Linear mixed effects models showed no significant difference between the models using scene cut information from V training sets compared to AV training sets (*estimate* = 1.166 × 10^−3^, *df* = 1388, *p* = 0.405). The Gabor only information had slightly better model performance in the V-only condition, however, this increase was not significant (*estimate* = 1.418 × 10^−3^, *df* = 1388, *p* = 0.448).

To understand whether the higher overall performance and cross-prediction performance in the visual models was driven by the input EEG data, we performed a direct correlation between the bandpass filtered EEG signals in the AV and A conditions and compared those to the correlation between the bandpass filtered EEG in the AV and V conditions. We found that EEG data in the occipital channels were more correlated for between AV and V than between AV and A conditions (Fig 3D). This suggests that the visual information has an outsized effect on the EEG itself in both visual and audiovisual conditions, although the weights are correlated between unimodal and multimodal conditions.

## Discussion

The use of naturalistic audiovisual stimuli in EEG research has become increasingly important in understanding the complex relationship between sensory modalities, while simultaneously providing a unique and valuable tool for investigating neural processing in a more ecologically valid context. Using stimuli that are more engaging to investigate neural responses to speech or visual information improves the participant’s experience with the experimental paradigm, leading them to feel less fatigued and discontent [2, 5]. Based on our prior findings from [9], we designed an experiment to identify if auditory and visual information were differently encoded in natural stimuli in three varying conditions: AV, V, and A. We show that AV training data can be used to derive both auditory and visual receptive fields that are comparable to those achieved using unimodal stimuli. In other words, if one were interested in using multimodal stimuli such as these movie stimuli, or other stimuli in which audiovisual features are relatively uncorrelated, it is possible to derive receptive fields to only auditory or only visual information that are similar to the unimodal condition. Our cross-prediction analysis demonstrated that we could independently predict responses to unimodal information even when using a naturalistic audiovisual stimulus set. While we found similar receptive field tuning for some electrodes when comparing A-only and AV and comparing V-only with AV, predicting neural responses to visual information was similarly robust when using AV training data versus V training data. In contrast, predicting neural responses to auditory information from the AV condition was possible but not as robust as the model performance for AV versus V data. The results from this study and previous work using these stimuli [9] showed that while it is possible to use an acoustically rich audiovisual stimulus such as this one to derive auditory receptive fields, the visual information does contain a large presence in the EEG signal. As such, the results from the cross-prediction analysis in Fig 3 demonstrate visual information has a relatively larger influence on the recorded EEG signal than auditory information when using multimodal naturalistic stimuli.

In previous work that examined sensory processing of audiovisual information, many included stimuli with face information and non-animated stimuli [17, 20–24]. Some of these studies showed that visual information can help disambiguate auditory information, particularly in the presence of noise. This is useful especially for people with hearing loss, who can rely on visual information to supplement auditory information as a way to improve speech comprehension and reduce listening effect [20, 21]. Additionally, these studies controlled for the visual and auditory information, respectively, allowing for the exploration of the effects of visual information on auditory and vice versa and the convergence of sensory modalities. In our study, we use movie trailers in their original format and separated the auditory and visual information. However, our main question was to investigate if auditory receptive fields could be derived despite the strong presence of visual information (as we demonstrated in our previous study [9]). If so, we argue that using naturalistic audiovisual stimuli such as these animated movie trailers still uncover tuning to auditory and visual stimuli, despite the presence of the opposite modality. While we saw little influence of visual information on the auditory information in our stimuli and vice versa, this is not to say that visual information is not helpful for improving speech comprehension. For stimuli with relatively more dependence between auditory and visual signals, unimodal and multimodal encoding may differ more than what we saw with our stimuli. Still, other researchers would be able to use similar methods to analyze the structure and feature correlations (e.g. in Fig 3B) to determine how appropriate or generalizable the derived receptive fields would be for their own stimuli. Another motivation for using movie trailers is to present stimuli that are more ecologically valid and engaging to participants. As we have found, the visual information does not significantly affect the ability to uncover neural responses to auditory information, providing evidence that these children’s movie trailers are a viable option for studying speech as well as visual information in healthy adults and ultimately would be useful when working with clinical and pediatric populations.

We provide motivation for replacing more controlled naturalistic speech-only experimental paradigms with tasks that include multisensory information and are better representative of speech encoding in our daily environment. Additionally, using AV stimuli allows for addressing multiple research questions with the same rich dataset.
